# Diffuse cauda equina nerve root enlargement: diagnostic challenges, clinicopathological spectrum, and the role of surgical biopsy

**DOI:** 10.3389/fneur.2026.1872205

**Published:** 2026-07-17

**Authors:** Chunli Lu, Xingwen Wang, Min Yin, Haipeng Si

**Affiliations:** 1 Department of Spine Surgery, Qilu Hospital of Shandong University (Qingdao), Cheeloo College of Medicine, Shandong University, Qingdao, Shandong, China; 2 Neurospine Center, China International Neuroscience Institute, Xuanwu Hospital, Capital Medical University, Beijing, China; 3 The Key Laboratory of Qingdao in Medicine and Engineering, Qilu Hospital of Shandong University (Qingdao), Shandong University, Shandong, China; 4 Department of Gynecology, Qilu Hospital of Shandong University (Qingdao), Cheeloo College of Medicine, Shandong University, Qingdao, Shandong, China

**Keywords:** chronic inflammatory demyelinating polyradiculoneuropathy, diagnostic challenge, diffuse cauda equina enlargement, neurolymphomatosis, surgical biopsy

## Abstract

**Backgrounds:**

Diffuse enlargement of the cauda equina nerve roots is an uncommon radiological finding that may result from various causes, including neoplastic, inflammatory, and immune-mediated conditions. Because these causes have overlapping imaging features, it remains challenging to differentiate among them, and effective diagnostic strategies are not well established.

**Purpose:**

To characterize the clinicoradiological features of patients presenting with diffuse cauda equina root enlargement and to evaluate the diagnostic role of surgical biopsy in cases with uncertain etiology.

**Methods:**

We retrospectively reviewed 12 patients presenting with symptoms of cauda equina and nerve root involvement, along with diffuse nerve root enlargement on MRI, between 2019 and 2024. Clinical presentation, imaging findings, diagnostic workup, treatment strategies, and outcomes were analyzed descriptively. Surgical decompression with biopsy was performed in selected patients with rapid neurological deterioration or unclear diagnosis.

**Results:**

All patients exhibited diffuse enlargement of the cauda equina nerve roots on MRI, although the underlying etiologies were diverse, including malignant lymphoma and immune-mediated neuropathies. In selected cases with diagnostic uncertainty, surgical biopsy provided important pathological information that facilitated a definitive diagnosis and subsequent therapy directed by the diagnosis. Patients with lymphoma generally demonstrated favorable responses to systemic chemotherapy, while those with immune-mediated disorders improved following immunotherapy. Clinical outcomes appeared to vary according to the underlying disease process rather than the extent of radiological abnormalities at presentation.

**Conclusion:**

Diffuse cauda equina nerve root enlargement represents a radiological pattern associated with heterogeneous etiologies rather than a single disease entity. Careful diagnostic evaluation is required, and surgical biopsy may be helpful in selected cases when noninvasive investigations remain inconclusive.

## 1 Introduction

Hypertrophic neuropathy is an uncommon cause of cauda equina dysfunction and may present with a broad spectrum of clinical manifestations, including progressive radiculopathy, sensory disturbances, motor weakness, and, in advanced stages, features of cauda equina syndrome (1). In clinical practice, diffuse enlargement of the cauda equina nerve roots on magnetic resonance imaging (MRI) is a rare but clinically significant radiological finding that represents a phenotype rather than a specific disease entity (2). This imaging pattern may arise from a wide range of pathological processes involving the peripheral nerves, spinal nerve roots, or leptomeninges. Among the reported etiologies, chronic inflammatory demyelinating polyradiculoneuropathy (CIDP) and neurolymphomatosis (NL) are among the most frequently recognized causes (3). Accurate differentiation between these entities remains difficult because of substantial overlap in both clinical presentation and radiological appearance. MRI findings, including nerve root thickening and contrast enhancement, are often nonspecific, while laboratory investigations and cerebrospinal fluid analyses may yield inconclusive results. Consequently, delayed or incorrect diagnosis may result in inappropriate treatment and irreversible neurological impairment. Given these diagnostic complexities, establishing the underlying etiology is essential for appropriate management. In selected patients with rapidly progressive neurological deficits or inconclusive non-invasive investigations, surgical exploration with biopsy may provide valuable pathological evidence to guide diagnosis and treatment. In the present study, we retrospectively reviewed a series of patients with diffuse cauda equina nerve root enlargement to characterize their clinicoradiological and pathological features, explore the associated diagnostic challenges, and evaluate the utility of surgical biopsy in facilitating diagnosis-directed management.

## 2 Methods

### 2.1 Study design and patient selection

This retrospective case series included patients presenting with symptoms and signs of cauda equina and lumbosacral nerve root involvement, together with diffuse enlargement of cauda equina nerve roots on MRI, at our institution between June 2019 and May 2024. Clinical manifestations ranged from chronic radiculopathy and sensory-motor deficits to, in some patients, bladder or bowel dysfunction consistent with cauda equina syndrome. Rather than representing a single disease entity, this radiological pattern was considered a shared manifestation of heterogeneous underlying conditions.

Inclusion criteria were (1): clinical evidence of cauda equina or lumbosacral nerve root dysfunction, including radicular pain, sensory disturbance, motor weakness, and/or sphincter dysfunction (2); diffuse nerve root enlargement involving ≥2 spinal segments on MRI; and (3) exclusion of traumatic, infectious, or purely degenerative etiologies. Patients with incomplete clinical or imaging data were excluded.

### 2.2 Diagnostic evaluation

All patients underwent a standardized diagnostic workup, including spinal MRI with and without contrast. Additional investigations, including cerebrospinal fluid (CSF) analysis, nerve conduction studies/electromyography (NCS/EMG), serological testing for autoimmune or paraneoplastic markers, and Fluorodeoxyglucose positron emission tomography/computed tomography (FDG-PET/CT), were performed when clinically indicated and available. Owing to clinical circumstances, patient condition, or technical limitations, not all patients underwent every ancillary examination.

In patients with rapid neurological deterioration or inconclusive non-invasive investigations, surgical exploration with biopsy was performed. The decision to proceed with surgery was made on a case-by-case basis, primarily for diagnostic clarification rather than for decompression alone. Resected specimens were subjected to histopathological and immunohistochemical analysis to establish a definitive diagnosis.

### 2.3 Treatment and follow-up

Subsequent treatment strategies were determined according to the underlying diagnosis, including chemotherapy for malignant conditions and immunotherapy for inflammatory or immune-mediated disorders. Clinical follow-up was conducted at 1, 3, and 6 months after discharge, with additional follow-up as needed. MRI was repeated during follow-up to assess radiological changes.

Clinical outcomes were descriptively categorized as improved, stable, or worsened based on changes in neurological symptoms compared with baseline. Given the retrospective nature of the study, standardized functional outcome scores were not consistently available.

### 2.4 Statistical analysis

Given the small sample size, the analysis was primarily descriptive. Continuous variables are presented as ranges or medians, and categorical variables as counts and proportions. No formal hypothesis testing or inferential statistical analysis was performed. Data analysis was conducted using GraphPad Prism (version 10.2.3).

## 3 Results

### 3.1 Clinical characteristics

A total of 12 patients (7 females and 5 males; age range, 30–62 years) were included. Patients presented with a spectrum of cauda equina and nerve root–related symptoms, including varying degrees of lower limb weakness, sensory disturbances, lumbosacral pain, and, in some cases, bladder or bowel dysfunction. MRI demonstrated diffuse enlargement of the cauda equina and/or lumbosacral nerve roots in all cases, with T2-weighted hyperintensity or isointensity and variable contrast enhancement. Nine patients had a chronic disease course (>6 months), whereas three patients (Cases 2, 10, and 12) presented with acute or subacute onset (≤6 months). Five patients (Cases 1, 2, 3, 5, and 9) underwent surgical exploration with biopsy due to rapid neurological progression or inconclusive non-invasive evaluation. Following diagnosis-directed treatment, most patients showed clinical improvement to varying degrees. However, residual neurological deficits persisted in several cases. Detailed clinical, radiological, and treatment characteristics are summarized in Table 1.

**Table 1:** Clinical characteristics and outcomes of patients with diffuse cauda equina nerve root enlargement.

Case	Age, gender	Presentation	Symptom duration	Diagnosis	Treatment	Follow-up period	Outcome
1	55, M	Lumbosacral pain, bilateral numbness	1 year	CIDP	Biopsy + steroids	2 years	Improved
2	54, F	Pain, weakness (rapid)	1 month	DLBCL	Biopsy + chemotherapy	5 years	Improved
3	30, F	Weakness, back pain	8 years	MS	Biopsy + immunotherapy	6 years	Improved
4	54, F	Numbness, gait disturbance	3 years	CIDP	Medical therapy	5 years	Stable
5	61, F	Leg numbness, bladder dysfunction	6 months	CIDP	Biopsy + steroids	3 years	Improved
6	40, M	Back pain, gait difficulty	1 year	IgG4-RD	Immunotherapy	4 years	Stable
7	52, F	Paresthesia, foot drop	3 years	CIDP	Steroids	5 years	Improved
8	36, M	Weakness, bowel dysfunction	2 years	CIDP	IVIG + steroids	4 years	Stable
9	58, F	Paraplegia, weight loss (rapid)	2 weeks	DLBCL	Biopsy + chemotherapy	1 year	Improved
10	62, M	Weakness (subacute)	2 months	DLBCL	Chemotherapy	3 years	Stable
11	45, F	Back pain, numbness	2 months	DLBCL	Chemotherapy	4 years	Stable
12	60, M	Acute retention, weakness	10 days	DLBCL	Chemo + RT	6 years	Worsened

M: male; F: female; CIDP: chronic inflammatory demyelinating polyradiculoneuropathy; DLBCL: diffuse large B-cell lymphoma; MS: multiple sclerosis; IgG4-RD: IgG4-related disease; RT: radiation.

### 3.2 Etiological classification with clinical and radiological features

Based on final diagnosis, patients were categorized into immune-mediated (*n* = 7) and neoplastic (*n* = 5) groups. The immune-mediated group included chronic inflammatory demyelinating polyradiculoneuropathy (CIDP, *n* = 5), multiple sclerosis (MS, *n* = 1), and IgG4-related disease (*n* = 1). A detailed summary of the diagnostic workup, including cerebrospinal fluid findings, electrophysiological studies, FDG-PET/CT results, serological investigations, biopsy status, and diagnostic criteria for each final diagnosis, is provided in Supplementary Table S1. These patients generally exhibited a more indolent course with gradually progressive neurological symptoms. MRI findings typically showed diffuse or patchy nerve root enlargement with T2 hyperintensity and mild to moderate contrast enhancement, without discrete mass formation. A detailed summary of the MRI findings is provided in Table 2.

**Table 2:** MRI Findings and enhancement characteristics of diffuse cauda equina enlargement.

Case	Neurological progression	Segments involved	Enhancement pattern+	Max diameter#
1	Chronic	>5	Diffuse	>6 mm
2	Rapid	>5	Diffuse	>6 mm
3	Chronic	>5	Diffuse	>6 mm
4	Chronic	>5	Not assessable	>6 mm
5	Chronic	3–5	Mild	4–6 mm
6	Chronic	>5	Diffuse	>6 mm
7	Chronic	3–5	Mild	4–6 mm
8	Chronic	>5	Not assessable	>6 mm
9	Rapid	<3	Diffuse	>6 mm
10	Rapid	3–5	Not available	>6 mm
11	Rapid	3–5	Not available	>6 mm
12	Rapid	>5	Diffuse	>6 mm

+For clarity, enhancement patterns were simplified into diffuse, mild, not assessable, and not available categories based on the overall extent of post-contrast nerve root enhancement.

#Max diameter of cauda equina nerve root.

The neoplastic group consisted of five patients with diffuse large B-cell lymphoma (DLBCL). These patients more frequently presented with subacute or rapidly progressive neurological deficits. MRI demonstrated diffuse nerve root enlargement with relatively homogeneous and marked enhancement. In selected cases, FDG-PET/CT showed increased metabolic activity corresponding to the affected regions. Despite overlapping imaging features, definitive differentiation between these etiologies required integration of clinical, laboratory, and histopathological findings.

### 3.3 Surgical findings and diagnostic yield

Five patients underwent laminectomy with intraoperative biopsy. The primary indication for surgery was diagnostic uncertainty or rapid neurological deterioration rather than radiologically confirmed mechanical compression alone.

Intraoperatively, varying degrees of nerve root enlargement were observed. Histopathological examination established definitive diagnoses in all operated cases, including CIDP, DLBCL, and MS. No immediate postoperative neurological deterioration was documented.

Following histopathological confirmation, all patients received diagnosis-directed therapy. Patients with immune-mediated conditions were treated with corticosteroids and/or intravenous immunoglobulin, while those with DLBCL received systemic chemotherapy. Clinical improvement was observed in several cases after initiation of targeted treatment.

### 3.4 Follow-up outcomes

The mean follow-up duration was 4 years (range, 1–6 years). At the last follow-up, most patients demonstrated either improvement or stabilization of neurological function. Based on overall clinical status, 6 patients (50%) were categorized as improved, 5 patients (41.7%) as stable, and 1 patient (8.3%) as worsened. Outcomes appeared to vary according to underlying etiology rather than initial radiological appearance. To provide a more objective assessment of outcomes, detailed longitudinal data regarding motor function, ambulation, pain severity, sensory symptoms, and bladder/bowel function are summarized in Supplementary Table S2.

### 3.5 Illustrative cases

#### 3.5.1 Case 1 (CIDP)

**Figure 1:**
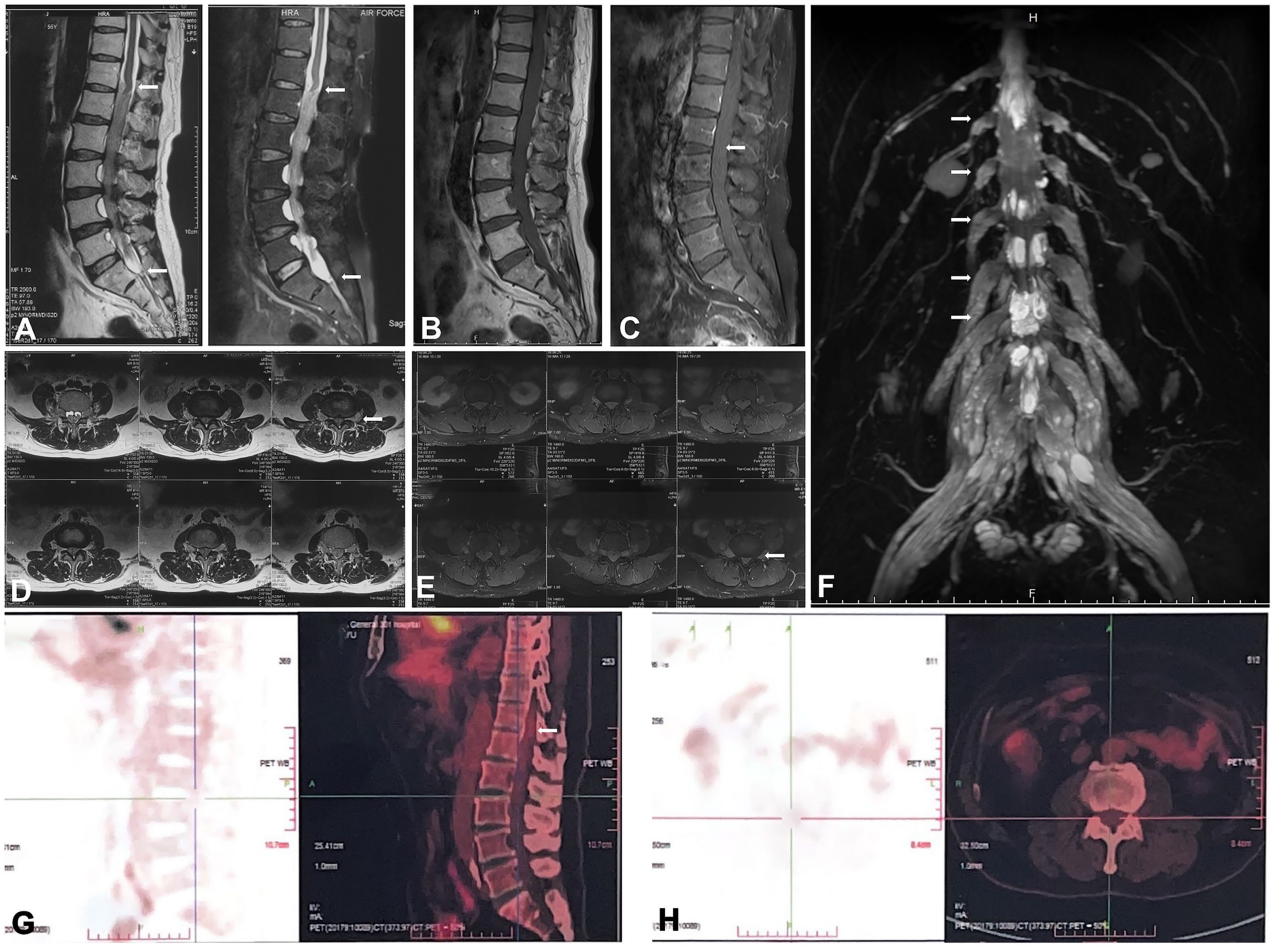
Illustrative Case 1. MRI findings demonstrating diffuse enlargement of the cauda equina and lumbosacral nerve roots from T12 to S1. Sagittal T2WI **(A)** and fat-suppressed T2WI **(B)** show extensive nerve root enlargement (arrows). Post-contrast T1-weighted imaging demonstrates partial enhancement (**C**, arrow). Coronal T2WI reveals bilateral hyperintense lesions involving multiple segments (**D**, arrows), with minimal enhancement on post-contrast imaging (**E**, arrows). Axial MRI shows diffusely enlarged nerve roots (**F**, arrows). PET/CT demonstrates enlargement of the neural foramina and mildly increased metabolic activity **(G,H)**.

**Figure 2:**
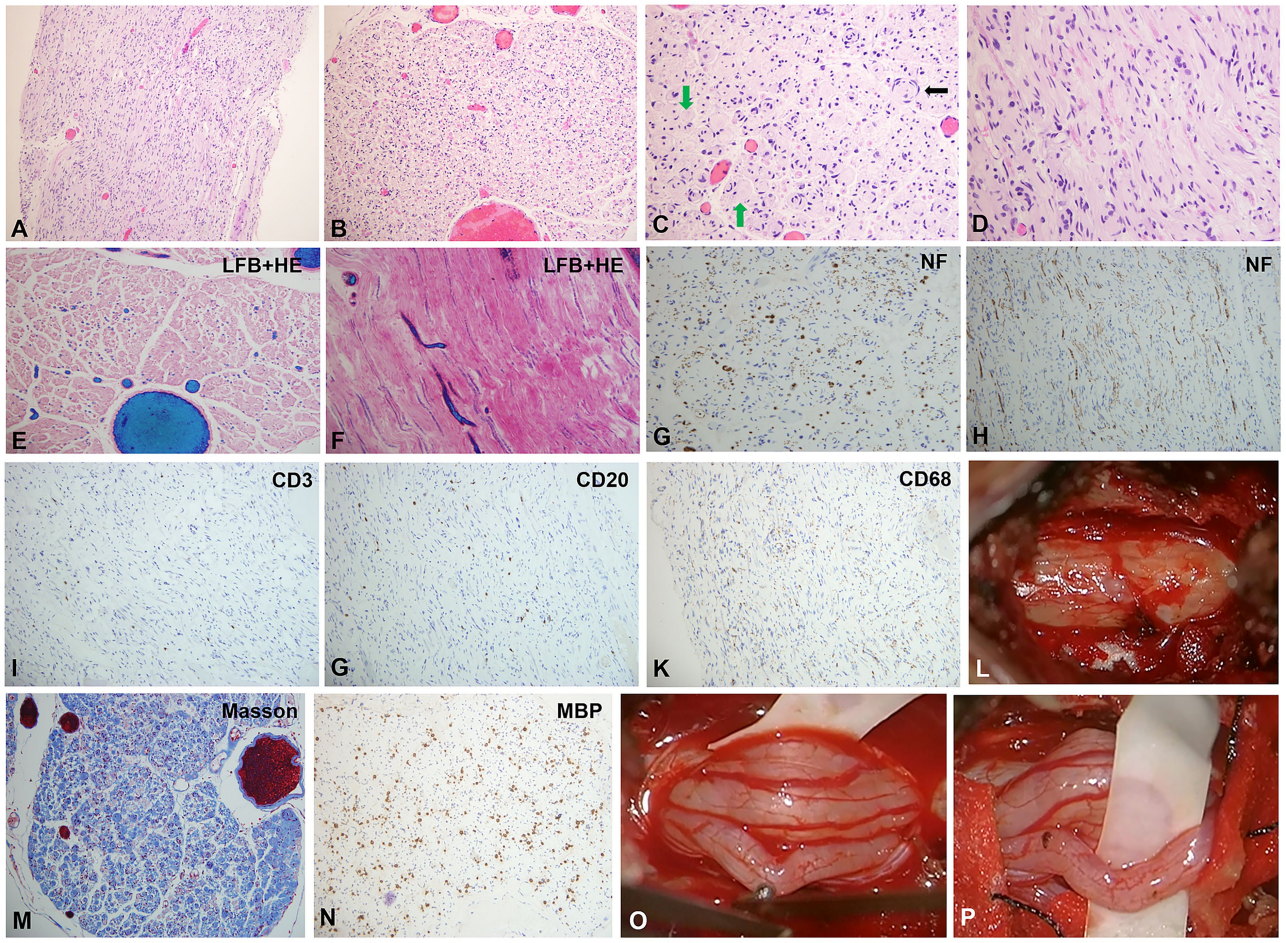
Histopathological findings of illustrative Case 1. Cross-sectional examination demonstrates moderate-to-severe demyelination with relative axonal preservation **(A–D)**. Focal axonal swelling, Schwann cell proliferation, and occasional onion-bulb formations are observed **(C)**. Interstitial vascular dilatation and scattered inflammatory cell infiltration are present. Immunohistochemical staining shows positivity for LFB + HE, NF, focal positivity for CD3, CD20, and CD68 **(E–K)** with Masson and MBP staining (M, N). Intraoperative views demonstrate enlargement of the cauda equina nerve roots **(L,O,P)**.

A 55-year-old man presented with chronic lumbosacral discomfort and progressive bilateral lower limb numbness. MRI revealed diffuse enlargement of the cauda equina nerve roots. Nerve conduction studies were consistent with demyelinating neuropathy. Due to diagnostic uncertainty, a surgical biopsy was performed, demonstrating demyelination with onion bulb formations, consistent with CIDP. The patient was treated with corticosteroids and showed gradual clinical improvement during follow-up (Figures 1, 2).

#### 3.5.2 Case 2 (DLBCL)

**Figure 3:**
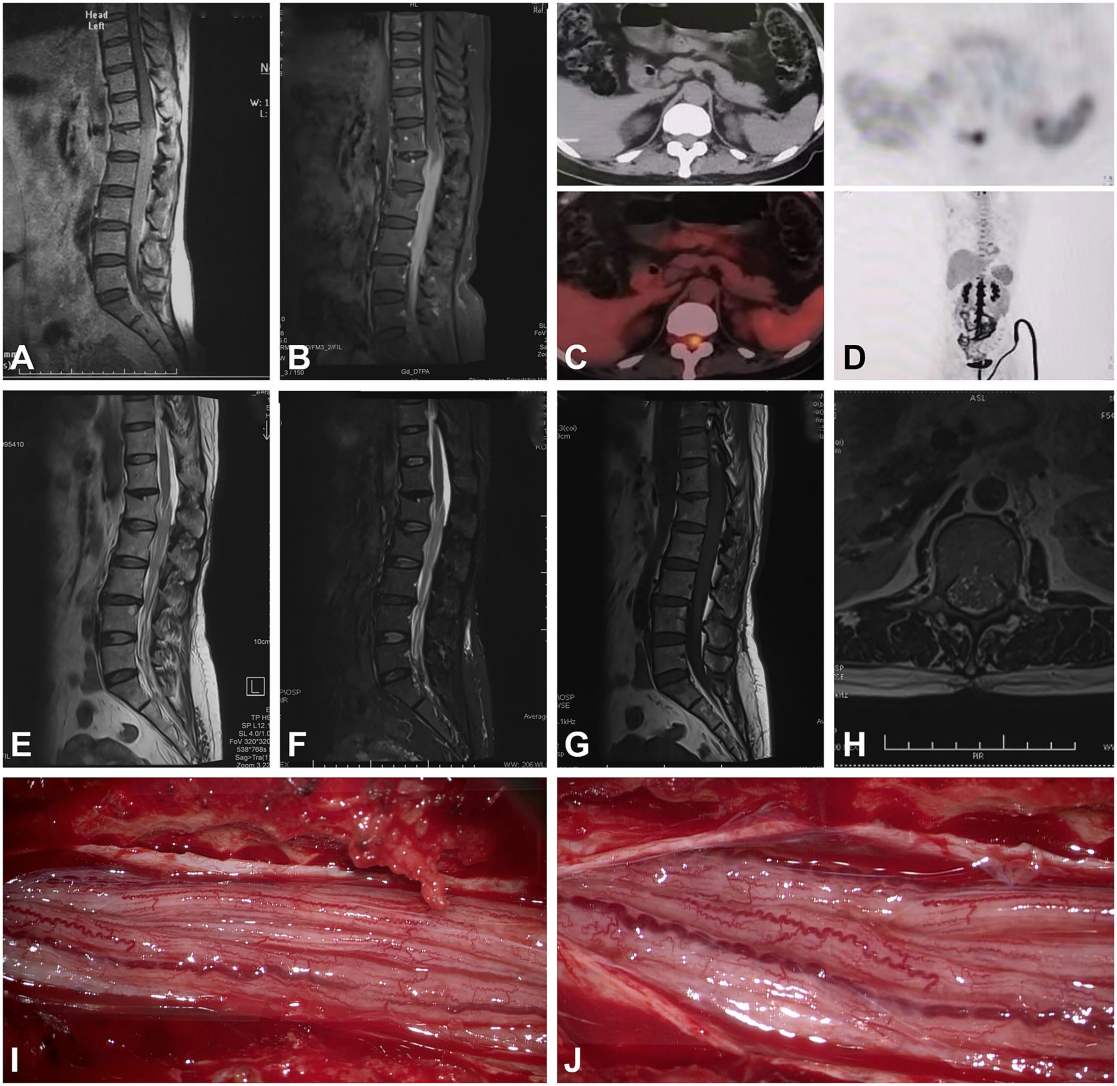
Illustrative Case 2. Lumbar MRI demonstrates diffuse enlargement of the cauda equina from L1 to S1. The lesions are isointense on T1-weighted imaging **(A)** and hypointense on T2-weighted imaging **(E–H)**. Contrast-enhanced T1-weighted imaging shows diffuse enhancement of the enlarged nerve roots **(B)**. FDG-PET demonstrates increased tracer uptake within the cauda equina **(C,D)**. Intraoperative findings reveal markedly enlarged cauda equina nerve roots with grayish infiltrative lesions adherent to the nerve roots **(I,J)**.

**Figure 4:**
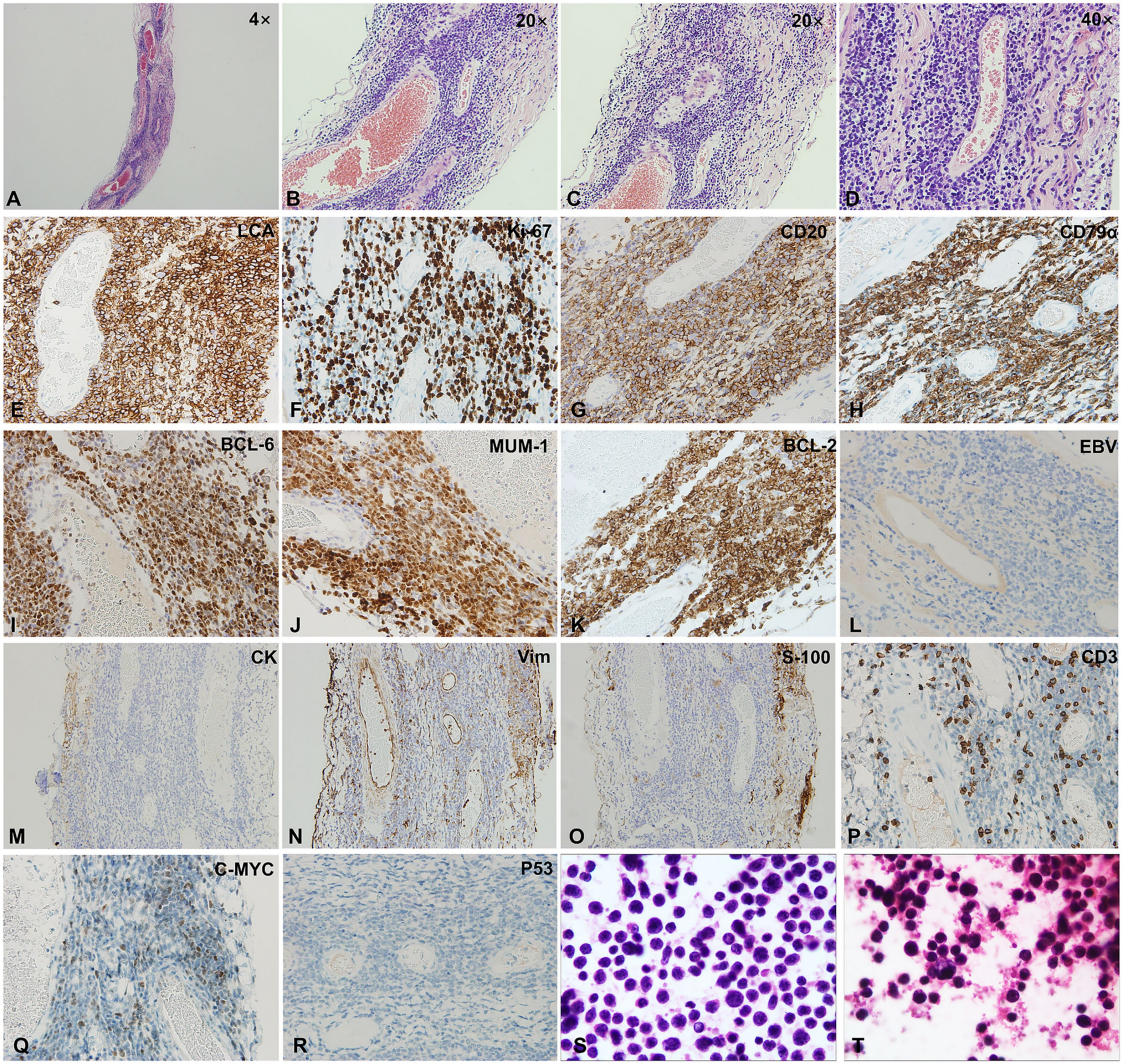
Pathological findings of Case 2. Histological examination demonstrates diffuse infiltration of atypical lymphoid cells within fibrous connective and peripheral nerve tissues **(A–D)**. Immunohistochemical staining shows positivity for LCA, Ki-67 (approximately 80%), CD20, CD79α, BCL6, MUM1, and BCL2 **(E–K)**, while EBV, CK, VIM, S100, CD3, c-Myc, and p53 are negative **(L–R)**. S100 staining highlights residual nerve tissue **(O)**. Cerebrospinal fluid cytology reveals atypical lymphoid cells with enlarged nuclei and occasional mitotic figures **(S,T)**.

A 54-year-old woman presented with rapidly progressive lower limb weakness and sensory deficits. MRI demonstrated diffuse cauda equina enlargement with marked contrast enhancement, and FDG-PET/CT showed increased metabolic activity. CSF analysis revealed pleocytosis and elevated protein levels. Surgical biopsy confirmed diffuse large B-cell lymphoma. The patient received systemic chemotherapy and achieved sustained clinical remission during long-term follow-up (Figures 3, 4).

## 4 Discussion

Diffuse enlargement of the cauda equina nerve roots is an uncommon but clinically important radiological finding. This distinction is critical, as interpretation of this entity as a unified disease may lead to diagnostic ambiguity and inappropriate management (1, 2). Rather than representing a distinct disease entity, our results support the concept that this pattern reflects a shared manifestation of heterogeneous underlying conditions (4), including immune-mediated neuropathies and malignant infiltration (5).

In the present series, CIDP and diffuse large B-cell lymphoma (DLBCL) represented the two most common etiologies, consistent with previous reports (6). Despite fundamentally different pathophysiological mechanisms, these conditions demonstrated overlapping clinical presentations and imaging features (7). Patients with CIDP typically showed a more indolent course and diffuse nerve root enlargement with mild to moderate enhancement, whereas those with lymphoma more often presented with rapid neurological deterioration and more homogeneous enhancement (7). However, these imaging differences lacked sufficient specificity to permit reliable etiological differentiation (8), underscoring the limitations of MRI as a standalone diagnostic tool (9).

This diagnostic uncertainty has important clinical implications (8). In our cohort, non-invasive investigations including MRI, cerebrospinal fluid analysis, and electrophysiological studies were frequently insufficient to establish a definitive diagnosis (10). Consequently, reliance on a presumptive diagnosis may either delay timely treatment of malignant disorders or expose patients with benign inflammatory conditions to unnecessary aggressive therapies (11).

In such circumstances, surgical biopsy may provide critical pathological information that facilitates accurate diagnosis and supports the selection of appropriate disease-specific treatment (12). Histopathological examination provided critical diagnostic information in the operated patients and facilitated subsequent diagnosis-directed treatment (13). Although surgical biopsy is inherently invasive and should not be considered a first-line diagnostic procedure, our findings suggest that it may have particular value in carefully selected patients with atypical clinical features, rapidly progressive neurological deficits, or inconclusive non-invasive investigations (14).

It is important to recognize that clinical outcomes in this series appeared to be influenced primarily by the underlying disease process rather than by surgical intervention itself (15, 16). Patients with lymphoma generally responded favorably to systemic chemotherapy, whereas those with immune-mediated disorders showed clinical improvement following immunotherapy (16). Consequently, surgical intervention could be viewed as a diagnostic adjunct that facilitates timely and appropriate disease-specific treatment rather than as a definitive therapeutic strategy (17). The overlap between immune-mediated and neoplastic processes further complicates clinical decision-making. In rare cases, lymphoma may present with neuropathic features mimicking inflammatory disease, and prolonged immunosuppressive therapy may obscure or delay the diagnosis of malignancy (18). These considerations underscore the importance of maintaining a broad differential diagnosis and a low threshold for tissue confirmation in atypical or rapidly progressive cases (19).

From a diagnostic standpoint, our findings support the concept that diffuse cauda equina nerve root enlargement should be regarded as a radiological phenotype rather than a distinct disease entity. Accurate diagnosis, therefore, requires a systematic etiological evaluation integrating clinical progression, imaging findings, cerebrospinal fluid analysis, electrophysiological studies, and, when necessary, histopathological assessment. Advanced imaging modalities such as FDG-PET/CT may provide complementary information in selected cases, particularly when malignancy is suspected; however, they cannot reliably replace tissue diagnosis when diagnostic uncertainty persists.

Several limitations should be acknowledged. First, the retrospective design and relatively small sample size limit the generalizability of our findings. Second, the heterogeneity of the underlying etiologies precluded meaningful statistical comparisons and restricted assessment of disease-specific prognostic factors. Third, standardized functional outcome measures were not uniformly available for all patients, and outcome assessment was therefore based primarily on retrospective clinical documentation. These limitations should be considered when interpreting the results.

Despite these limitations, this study provides additional insight into a rare and diagnostically challenging clinical presentation. Our findings emphasize that diffuse cauda equina nerve root enlargement encompasses a heterogeneous spectrum of disorders and suggest that surgical biopsy may provide valuable diagnostic information in carefully selected patients with unresolved diagnostic uncertainty. Future multicenter studies with larger cohorts are warranted to further refine diagnostic algorithms and better define the role of surgical intervention in this patient population.

## 5 Conclusion

Diffuse cauda equina nerve root enlargement represents a radiological pattern associated with heterogeneous etiologies. Comprehensive diagnostic evaluation is essential for accurate diagnosis, while surgical biopsy may provide valuable pathological information in selected patients with persistent diagnostic uncertainty. Treatment strategies and outcomes are largely determined by the underlying etiology.

## Data Availability

The raw data supporting the conclusions of this article will be made available by the authors, without undue reservation.
