# Supplementary material for: Diffuse cauda equina nerve root enlargement: diagnostic challenges, clinicopathological spectrum, and the role of surgical biopsy
Source: Front Neurol. 2026 Jul 17;17:1872205. doi: 10.3389/fneur.2026.1872205 (PMC13423981; doi:10.3389/fneur.2026.1872205)
Supplement: Supplementary file 1 [file Table_1.DOCX]

**Case 3.**

A 30-year-old female presented with recurrent weakness in both lower extremities for 8 years and weakness in all four extremities for 33 months. She was treated with intravenous immunoglobulins for 5 days at a local hospital, and the symptoms improved and numbness disappeared. The results of electrodiagnostic examination indicated peripheral neurogenic damage in the upper and lower extremities. At this point, lumbosacral MRI with and without demonstrated significant enlargement of the cauda equina and lumbosacral plexus nerves at the level of the lumbosacral vertebral body with an enhancement, progressive compared to the previous lesion, and multiple abnormal signal shadows in the lumbosacral foramen and pelvic plexus (Fig S1A-D). With existing weakness in the extremities, the patient was advised to undergo neurosurgery. CSF analysis revealed a notably elevated white blood cell (WBC) count (21×10^6^/ L), significantly elevated protein level (435 mg/dL), and normal glucose level. A biopsy of cauda equina neuropathy was performed under general anesthesia, and an enlarged cauda equina was observed (Fig S1E and F). Histochemical staining demonstrated a marked enlargement of the nerve fibers, with marked loss of myelin sheath and axonal lesion, Schwann cell hyperplasia partially arranged in a swirling pattern, interstitial small vessel hyperplasia with mucus-like degeneration, and scattered T cell-dominated lymphocyte and phagocytic infiltration in the interstitium (Fig S1G-L). Immunohistochemistry revealed that LFB+HE (+), NF(+), MBP(+), CD20(+), CD68(+), and CD8(+) (Fig S1M-T). CIDP combined with Multiple sclerosis (MS) was considered, and the patient was discharged after administration of steroids and symptomatic supportive therapy. The patient was treated with plasma exchange five times, each time with a plasma volume of approximately 2300-2400 ml, without transfusion reactions and complaints of discomfort, after which the symptoms could be relieved. 48 months ago, the patient had received rituximab (Merova) and reported improvement in limb weakness and peripheral pain. He was administered anticoagulant therapy and continued to use Merova, and the pain in the lower extremities and whole body was reduced.


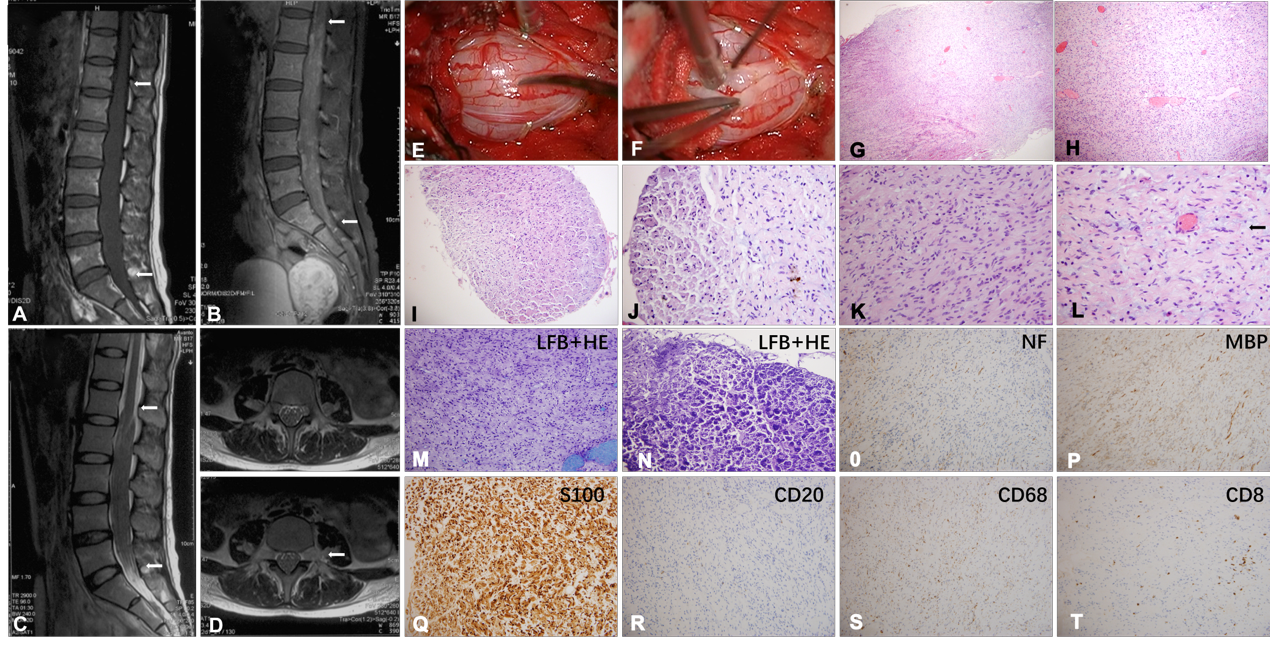


**Supplementary Fig. 1 Illustrative Case 3.** Significant enlargement of the cauda equina and lumbosacral plexus nerves at the level of the lumbosacral vertebral, hypointense in T1WI (A, between white arrows), hyperintense in T2WI (C, between white arrows; D, white arrow), and partial hyperintense lesions in T1 enhancer (B, between white arrows). Histochemical stain demonstrated showed marked enlargement of the nerve fibers(G), with marked loss of myelin sheath and axonal impairment, with Schwann cell hyperplasia, partially arranged in a swirling pattern, interstitial small vessel hyperplasia (H) with scattered T cell-dominated lymphocyte and phagocytic infiltration in the interstitium (K), and mucus-like degeneration (L, black arrow). Immunohistochemistry released LFB+HE (+), NF(+), MBP(+), CD20(+), CD68(+), and CD8(+). (M-T).

**Case 4.**

A 54-year-old female presented with numbness and weakness of limbs for 3 years, unsteady walking. Recent occasional urinary incontinence and constipation. On examination she was neurologically intact apart from some slightly decreased pain and temperature sensation in the area below the groin of the lower extremities. MRI scan revealed the hypertrophic spinal nerve roots (Fig S2A-B). Macroscopically the lesion involved multiple nerve roots that expanded into enlarged fascicles. At the same time, lumbar puncture, lumbosacral MRI, PET-CT and other examinations were improved, and the results did not indicate the presence of tumor infiltration, this case could preferably be regarded as a hypertrophic neuropathy of the cauda equina. Proximal both lower extremity is grade 3, and distal right lower extremity is grade 0 with decreased knee jerk reflex. Past history: previous history of polio; previous history of hypertension, diabetes, hyperlipidemia; previous history of trauma surgery on the left lower extremity. She suffered numbness and weakness of lower limbs, and bladder and bowel dysfunction. Dexamethasone 40mg intravenous infusion for 6 days and then switch to oral corticosteroids. After 3 months, the symptoms improved significantly.


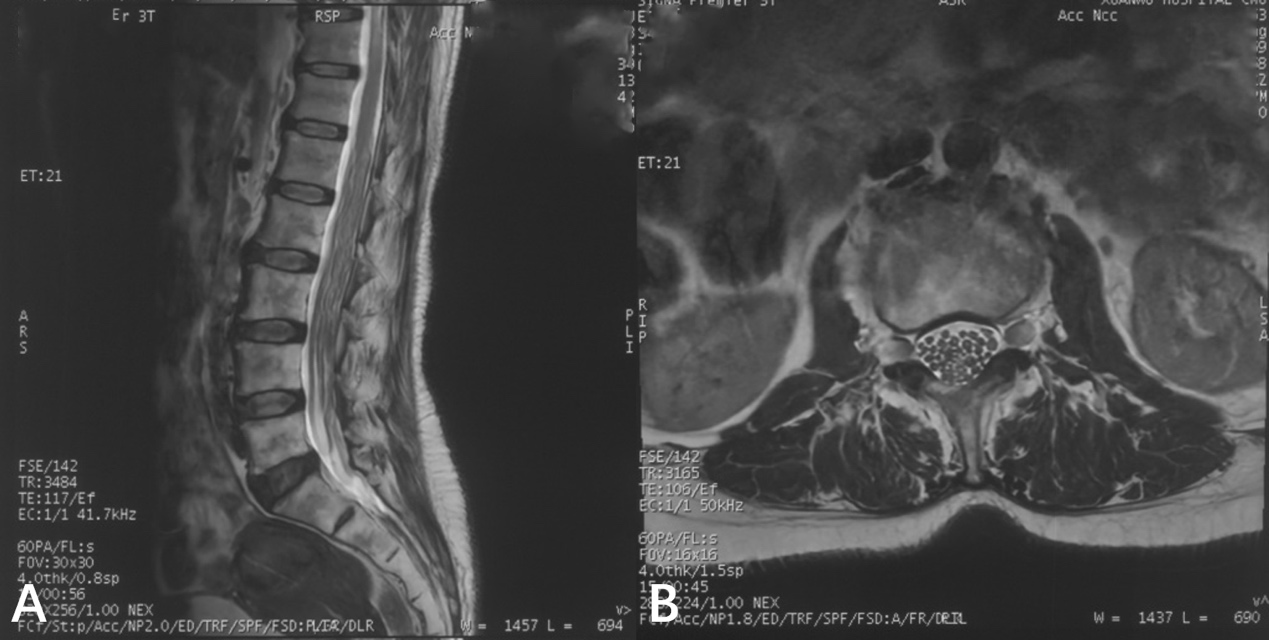


**Supplementary Fig. 2 Illustrative Case 4.** MRI of the lumbar spine showed swollen cauda equina occupying the dura sac from the L1-S1 level that was hypointense on T2WI (A, B).
