# Supplementary material for: Diffuse cauda equina nerve root enlargement: diagnostic challenges, clinicopathological spectrum, and the role of surgical biopsy
Source: Front Neurol. 2026 Jul 17;17:1872205. doi: 10.3389/fneur.2026.1872205 (PMC13423981; doi:10.3389/fneur.2026.1872205)
Supplement: Supplementary file 2 [file Table_2.DOCX]

**Supplementary Table S1: Standardized Diagnostic Workup and Definitive Diagnostic Basis**

| **Case** | **Motor Function (Objective Muscle Strength Grade)** | **Ambulation Capacity (Objective Description)** | **Pain Severity (Baseline vs Last Follow-up NRS Score)** | **Sensory Abnormalities** | **Bladder & Bowel Sphincter Function** | **Overall Outcome Grade** |
| --- | --- | --- | --- | --- | --- | --- |
| 1 | Bilateral lower limb strength improved from Grade 3 to Grade 4–5 (≥1 grade gain) | Fully independent level walking without limp | Baseline NRS=6; Final NRS=2 (significant reduction) | 70% reduction in the distribution of bilateral lower limb numbness and paresthesia | Complete voluntary voiding and defecation; no incontinence | Improved |
| 2 | Bilateral lower limb strength improved from Grade 2 to Grade 4, prominent proximal muscle recovery | Independent long-distance walking without orthotic support | Baseline NRS=7; Final NRS=1; resting radicular pain fully resolved | Distal lower limb proprioceptive deficits largely resolved | Previous urinary hesitancy and constipation completely resolved | Improved |
| 3 | Four-limb strength improved from Grade 2–3 to Grade 4 with balanced recovery | Independent flat-ground walking; mild fatigue climbing stairs | Baseline NRS=5; Final NRS=4 (no progressive worsening) | 50% reduction in the area of limb numbness | Normal voluntary bladder and bowel control; no incontinence | Improved |
| 4 | Sustained Grade 3 four-limb strength without improvement or decline | Independent short-distance walking; fatigable with prolonged activity | Baseline NRS=4; Final NRS=4 (no change) | Unchanged hypoesthesia below inguinal creases | Occasional mild stress urinary incontinence, no disease progression | Stable |
| 5 | Lower limb strength improved from Grade 3 to Grade 4–5 | Fully unassisted walking with normalized gait | Baseline NRS=6; Final NRS=2 | Marked alleviation of bilateral lower limb numbness and dysesthesia | Complete resolution of prior urinary retention and constipation | Improved |
| 6 | Persistent Grade 3–4 limb strength with no significant change | Independent flat-ground walking; bilateral limb soreness after prolonged ambulation | Baseline NRS=4; Final NRS=4 | Stable numbness distribution without new sensory deficits | Intermittent mild defecation difficulty; no incontinence | Stable |
| 7 | Foot drop strength recovered from Grade 2 to Grade 4 | Unassisted walking with resolution of foot drop and steppage gait | Baseline NRS=5; Final NRS=2 | Near-complete resolution of plantar numbness and paresthesia | Fully preserved voluntary sphincter function | Improved |
| 8 | Static Grade 3 four-limb strength with no recovery gains | Indoor ambulation requires cane; unable to complete long-distance walking | Baseline NRS=4; Final NRS=4 | Persistent diffuse limb numbness with no progression | Intermittent incomplete bladder emptying, no worsening | Stable |
| 9 | Bilateral lower limb strength improved from Grade 1 (near paraplegia) to Grade 3 | Indoor ambulation with walker support; short-distance independent outdoor walking | Baseline NRS=8; Final NRS=3 | Marked relief of lower limb allodynia | Significant improvement in urinary retention and incontinence | Improved |
| 10 | Persistent Grade 3 lower limb strength with no recovery | Daily walking requires crutch assistance; unable to climb stairs | Baseline NRS=5; Final NRS=5 | Persistent lumbosacral numbness without new lesions | Occasional urinary hesitancy, no progressive dysfunction | Stable |
| 11 | Stable Grade 4 four-limb strength without fluctuation | Fully independent flat-ground walking with normal gait | Baseline NRS=3; Final NRS=3 | Static lumbosacral paresthesia with no aggravation | Fully intact bladder and bowel function | Stable |
| 12 | Lower limb strength deteriorated from Grade 3 to Grade 0–1 with severe bilateral weakness | Full-time wheelchair dependence; unable to stand unsupported | Baseline NRS=4; Final NRS=7 with intractable persistent radicular pain | Permanent complete lower limb numbness plus progressive loss of deep proprioception | Refractory continuous urinary and fecal incontinence without voluntary control | Worsened |

Abbreviations: NR = Not retrospectively retrievable; EFNS/PNS = European Academy of Neurology/Peripheral Nerve Society diagnostic criteria for CIDP; McDonald criteria = McDonald diagnostic criteria for multiple sclerosis (MS); DLBCL = Diffuse large B-cell lymphoma, confirmed via histopathology or systemic hematological workup
