# Supplementary material for: Diffuse cauda equina nerve root enlargement: diagnostic challenges, clinicopathological spectrum, and the role of surgical biopsy
Source: Front Neurol. 2026 Jul 17;17:1872205. doi: 10.3389/fneur.2026.1872205 (PMC13423981; doi:10.3389/fneur.2026.1872205)
Supplement: Supplementary file 3 [file Table_3.DOCX]

**Supplementary Table S2: Objective Functional Outcome Stratification**

| **Case** | **CSF Findings** | **EMG/NCS Findings** | **FDG-PET/CT Results** | **Serology Panel Tests** | **Biopsy Performed** | **Diagnostic Basis for Final Diagnosis** |
| --- | --- | --- | --- | --- | --- | --- |
| 1 | Elevated total protein; no malignant cells identified | Electrophysiological features consistent with demyelinating polyneuropathy | Mild radiotracer uptake within enlarged lumbosacral nerve roots | Negative autoimmune screening panel | Yes | Satisfied EFNS/PNS CIDP diagnostic criteria; intraoperative nerve root biopsy confirmed inflammatory demyelination with onion bulb formation |
| 2 | Cytosis with elevated CSF protein | NR | Intense hypermetabolism involving diffuse cauda equina lesions | Negative autoantibody and paraneoplastic serologies | Yes | Histopathological and immunohistochemical analysis of nerve biopsy specimen confirmed DLBCL |
| 3 | NR | NR | NR | NR | Yes | Met McDonald MS diagnostic criteria; nerve root biopsy revealed combined demyelination and T-cell predominant lymphocytic infiltration, consistent with concurrent CIDP and MS |
| 4 | Mildly elevated CSF protein | Electrophysiological demyelinating changes of bilateral lower extremities | No pathological hypermetabolic lesions | Negative autoimmune serology | No | Fully met clinical, electrophysiological and MRI EFNS/PNS CIDP criteria; PET excluded neoplastic infiltration |
| 5 | Elevated CSF protein | Bilateral lumbosacral polyradiculoneuropathy with demyelinating patterns | No abnormal hypermetabolic foci | Complete negative autoimmune panel | Yes | EFNS/PNS CIDP criteria supported by pathological demyelination on surgical biopsy |
| 6 | Mild elevation of CSF protein | NR | Heterogeneous mild metabolic uptake along affected nerve plexuses | Markedly elevated serum IgG4 concentration | No | Fulfilled comprehensive consensus diagnostic criteria for IgG4-related disease (serology, imaging and systemic clinical manifestations) |
| 7 | Elevated CSF protein | Multifocal demyelinating polyradiculopathy on neurophysiology | No tumor-like hypermetabolic lesions | Negative autoantibody screening | No | Met full EFNS/PNS clinical, electrophysiological and MRI diagnostic criteria for CIDP |
| 8 | Elevated CSF protein | Widespread peripheral nerve demyelination on EMG/NCS | No pathological radiotracer uptake | Negative autoimmune serology | No | Consistent with EFNS/PNS CIDP criteria; clinical response to immunotherapy corroborated diagnosis |
| 9 | Elevated CSF protein; atypical lymphoid cells detected on cytology | NR | Diffuse intense hypermetabolism of entire cauda equina | Negative paraneoplastic and autoimmune serologies | Yes | Surgical nerve root biopsy with immunohistochemistry confirmed DLBCL infiltration |
| 10 | Elevated CSF protein | NR | Hypermetabolic lesions along lumbosacral nerve roots | No specific abnormal serological markers | No | Combined systemic hematological evaluation and FDG-PET hypermetabolic imaging for clinical diagnosis of DLBCL neurolymphomatosis |
| 11 | Elevated CSF protein | NR | Multiple hypermetabolic foci within cauda equina | Negative lymphoma and autoimmune serology panels | No | Clinical diagnosis of DLBCL based on bone marrow workup and PET-CT whole-body imaging |
| 12 | Elevated CSF protein | NR | Diffuse hypermetabolism of full-length cauda equina | Unremarkable serological profiles | No | Systemic hematological assessment plus PET-CT hypermetabolic signal confirmed cauda equina DLBCL |

Standardized objective grading definitions: Improved: ≥30% reduction in symptomatic burden; ≥1 grade increase in motor strength; independent ambulation without assistive devices; ≥2-point drop in NRS pain score; full recovery of voluntary bladder/bowel control; Stable: All functional metrics fluctuate <10%; no meaningful deterioration or improvement in motor power, mobility, pain, sensation or sphincter function; Worsened: ≥1 grade decline in motor strength; wheelchair-dependent ambulation; progressive refractory pain; persistent irreversible bladder/bowel incontinence.
